# An Improved Migratory Birds Optimization Algorithm for Closed- Loop Supply Chain Network Planning in a Fuzzy Environment

**DOI:** 10.1371/journal.pone.0306294

**Published:** 2024-06-27

**Authors:** Yangjun Ren, Qiong Chen, Yui-yip Lau, Maxim A. Dulebenets, Mengchi Li, Botang Li, Mark Ching-Pong Poo, Pengfei Zhang

**Affiliations:** 1 School of Economics and Management, Changzhou Vocational Institute of Textile and Garment, Changzhou, China; 2 Navigation College, Jimei University, Xiamen, China; 3 Division of Business and Hospitality Management, College of Professional and Continuing Education, The Hong Kong Polytechnic University, Hong Kong, China; 4 Department of Civil and Environmental Engineering, Florida A&M University-Florida State University, Tallahassee, FL, United States of America; 5 School of Shipping Economics and Trade, Guangzhou Maritime University, Guangzhou, China; 6 Department of Port & Shipping Management, Guangzhou Maritime University, Guangzhou, China; 7 Liverpool Hope Business School, Liverpool Hope University, Liverpool, United Kingdom; 8 Liverpool Logistics, Offshore and Marine Research Institute, Liverpool John Moores University, Liverpool, United Kingdom; University of Isfahan, ISLAMIC REPUBLIC OF IRAN

## Abstract

Recycling of used products can provide substantial economic and environmental benefits for supply chain players. However, many factors associated with the design of closed-loop supply chain networks are uncertain in their nature, including demand, opening cost of facilities, capacity of opened facilities, transportation cost, and procurement cost. Therefore, this study proposes a novel fuzzy programming model for closed-loop supply chain network design, which directly relies on the fuzzy ranking method based on a credibility measure. The objective of the presented optimization model aims at minimizing the total cost of the network when selecting the facility locations and transportation routes between the nodes of the network. Based on the problem characteristics, a Migratory Birds Optimization Algorithm with a new product source encoding scheme is developed as a solution approach. The inspiration for the product source coding method originates from the label information of raw material supplier and manufacturing factories on product packaging, as well as the information of each logistics node on the delivery order. This novel encoding method aims to address the limitations of four traditional encoding methods: Prüfer number based encoding, spanning tree based encoding, forest data structure based encoding, and priority based encoding, thereby increasing the likelihood of heuristic algorithms finding the optimal solution. Thirty-five illustrative examples are developed to evaluate the proposed algorithm against the exact optimization method (LINGO) and a Genetic Algorithm, Ant Colony Optimization, Simulated Annealing, which are recognized as well-known metaheuristic algorithms. The results from extensive experiments show that the proposed algorithm is able to provide optimal and good-quality solutions within acceptable computational time even for large-scale numerical examples. The suitability of the model is confirmed through a meticulous sensitivity analysis. This analysis involves adjusting the confidence level incrementally from 50% to 100%, in 5% intervals, with respect to the model’s uncertain parameters. Consequently, it yields valuable managerial insights. The outcomes of this research are expected to provide scientific support for related supply chain enterprises and stakeholders.

## Section 1: Introduction

In recent years, energy shortages and environmental pollution have become the focus of global attention. The development of economic models charaterized by low energy consumption and low emissions is becoming a common choice for economic development in various countries worldwide. The logistics industry, being an energy-consuming industry and a major emitter of pollutants, makers it necessary to consider reducing carbon emissions when designing supply chain networks [1].

The closed-loop supply chain is valued by many countries due to its ability to reduce environmental pollution and conserve natural resources. It is composed of a forward supply chain network and a reverse supply chain network. In a forward supply chain network, suppliers provide raw materials to factories, and the products manufactured by these factories are transported to consumption areas via distribution centers. In the reverse supply chain network, waste or substandard products are recycled from the consumption areas back to the recycling center. Products that cannot be recycled undergo pollution-free treatment at the processing center, while the rest are disassembled at the disassembly center and transformed into raw materials for reuse in the factories. The key points of this entire process lie in the consumption area, recycling center, disassembly center, and factory, as these processes contribute to the coordination and integration of the forward and reverse supply network. Hence, the main scientific focus of this study is to integrate the forward and reverse supply chain network to form a closed-loop structure, which would significantly reduce the disposal of customer waste and old products, thereby substantialy mitigating environmental pollution.

In a closed-loop supply chain network, the key lies in understanding the reverse supply chain. Reverse supply chain networks can be divided into direct reusable networks, repair networks, recycling networks, and remanufacturing networks [2]. Reusable networks involve products that are directly reused, such as soft drink bottles, pallets, or containers. Repairing the networks focus on restoring faulty products to their working state, such as refurbishing household appliances like washing machines, electrical equipment, refrigerators, etc. Recycling networks means that materials are recycled without changing the product structure, such as steel products, glass, paper, etc. Remanufacturing networks involve transforming products into their original shape through operations such as disassembly, major repairs, cleaning, and replacement—for example, remanufacturing internal mechanical components like airplanes and automobiles. The closed-loop supply chain network model in this study demonstrates a general structure that can be utilized for recycling and processing processes. It is applicable to various industries, such as automotive engines, tires, and glass manufacturing.

The closed-loop supply chain network serves the purpose of reducing the utilization of raw materials, mitigating environmental pollution, and attaining social responsibility. As a result, it has emerged as a prominent research subject amongst numerous scholars [3]. The design of a closed-loop supply chain network presents a strategic optimization problem that entails determining the quantity of various facilities within the network, as well as the transportation volume between them, all with the goal of meeting customer needs while minimize overall costs. In a closed-loop supply chain network, the decisions regarding the location of each facility significantly impact the remanufacturing process. Furthermore, the optimal design of the network necessitates decides concerning the location and quantity of intermediate facilities, as the willingness of customers to return used products for remanufacturing varies across different regions. Consequently, the location decisions of different facilities hold great significance in the network design and optimization challenges faced by various remanufacturing industries. Additionally, when establishing a network, factors such as facility capacity limitations related to customers must be taken into account, as these can influence the level of service provided to customers and the number of returns collected from different customers.

In the real-world context, closed-loop supply chains exhibit a high degree of uncertainty [4]. Firstly, the total cost of the network includes fixed costs, operating costs within facilities, procurement costs, collection costs, and transportation costs. Due to differences in economic levels, levels of human resource, policies, and congestion levels of different plots and roads within the same region, the uncertainty of these costs exists. Managers of remanufacturing enterprises need to determine the level of risk based on market conditions. Additionally, the distribution of residents can affect the capacity of network facilities, such as irritating odors or sewage during the operation of dismantling centers and treatment centers, which limit facility capacity. Managers also need to take into account their risk situation. Furthermore, due to factors such as usage habits and climate in different regions affecting the quantity and quality of recycled products, they affect the overall recycling and remanufacturing rates. Finally, the greatest uncertainty in a closed-loop supply chain lies in demand, which can be influenced by internal and external factors such as economic conditions, personal income, and seasons. Therefore, when making strategic decisions on closed-loop supply chain networks, the credibility level of the aforementioned uncertainty situations should be considered. This is a problem that current research worth considering.

The closed-loop supply chain network problem belongs to a NP aid problem of combinatorial optimization [5]. For large-scale cases, utilizing an accurate method to solve them can result in long time calculation times. To address this challenge, researchers have turned to metaheuristic algorithms for optimizing closed-loop supply chain network. Heuristic algorithms offer the advantage of providing suboptimal solutions close to the global optimal solution within an acceptable time range. Currently, a growing number of heuristic algorithms have been successfully applied to closed-loop supply chain network design problems. While the processes of these heuristic algorithms may vary, their solution encoding methods share similarities. Notably, the encoding method employed in heuristic algorithms can significantly influence their efficiency. Regarding closed-loop supply chain network design problems, literature predeminantly adopts the following encoding methods: Prüfer number based encoding, spanning tree based encoding, forest data structure based encoding, and priority based encoding. Based on an extensive literature review and algorithm reproduction, it has been observed that despite the speed and efficiency advantages of these four encoding methods, heuristic algorithms utilizing them fail to seek the solution for certain special problems (even small-scale problems). As an example, utilizing the widely recognized priority-based coding method, Table 1 represents the current transportation problem’s optimal solution, while Table 2 represents the transportation volume obtained using priority based coding method. It is evident that regardless of changes in the priority order of suppliers and demand points, the optimal traffic volume allocation results shown in Table 1 cannot be obtained. Therefore, this study introduces a novel encoding method for solving the closed-loop network optimization problem, which has not been used in existing literature.

**Table 1:** Optimal traffic volume.

	Demand point (demand quantity)		
Supplier (capability)	1 (150)	2 (200)	3 (120)
1 (200)		100	19
2 (150)	150		
3 (100)			101
4 (250)		100	

**Table 2:** Traffic volume allocation based on priority encoding rules.

	Priority of demand points	3	2	1
		Demand point (demand quantity)		
Priority of suppliers	Supplier (capability)	1 (150)	2 (200)	3 (120)
2	1 (200)			70
4	2 (150)	150		
1	3 (100)			
3	4 (250)		200	50

The current research focus is to establish a closed-loop supply chain network model, considering the uncertainty of multiple parameters. The main problem that current research attempts to address is:

- How to establish a closed-loop supply chain network model considering multi parameter uncertainty?
- How to use a new meta heuristic algorithm to solve it?

The current research objectives are:

- Determine the optimal location of different facilities in the closed-loop supply chain network.
- Optimize and determine the transportation quantity between different facilities in the closed-loop supply chain network.
- Simultaneously consider fixed open costs, facility operating costs, procurement costs, collection costs, transportation costs, facility capacity constraints, and some additional constraints in the closed-loop supply chain.
- Using a fuzzy programming method based on credibility measure and fuzzy ranking method to deal with uncertainties in the model and address existing risks.
- Develop a new migratory bird optimization algorithm based on product source coding to search for the optimal solution considering closed-loop supply chain network optimization problems.

The structure of this research paper is as follows: Section 2 introduces a literature review on closed-loop supply chain problems. Section 3 shows the mathematical model of the closed-loop supply chain network problem under consideration. Section 4 provides a detailed explanation of the proposed new solution method for the migratory bird optimization algorithm based on product source coding. Section 5 encompasses a numerical experiment, using 35 examples generated from real data ranges with different scales to test the efficiency of the proposed algorithm. This section also includes sensitivity analysis on changes in uncertain parameters. Finally, management insights are proposed. Section 6 presents the conclusions of current research and future studies.

## Section 2: Literature review

A closed-loop supply chain is an integrated network including forward and reverse supply chains. Design of each closed-loop supply chain generally involves strategic decisions on the location, quantity, and capacity of facilities, transportation routes between nodes, purchase volumes, production volumes, and inventory [6]. In recent years, the economic benefits and environmental impacts brought by the recycling of used products have been increasing, prompting enterprises to pay more attention to the design of closed-loop supply chain networks [7–9]. In order to meet the established business goals in a competitive environment, enterprises have to accelerate recovery, recycling, remanufacturing, and disposal activities in the closed-loop supply chain network [1]. As such, finding a robust, scientific, resilient, and efficient closed-loop supply chain network design becomes crucial to solving the problem.

As the integration of forward and reverse supply chains directly influences the cost, service level, and operational environment, various researchers [10–13] conducted extensive research studies relevant to the design of closed-loop supply chain networks. Fleischmann et al. [2] first and systematically elaborated on the application of planning models in reverse logistics management and presented different types of network models. Fleischmann et al. [14] further developed a general model for closed-loop supply chain network design. The study indicated that due to the integration of forward and reverse network activities, a considerable reduction in production and operating costs, including the operating costs of warehouses and factories, could be achieved. Üster et al. [15] focused on minimizing the processing cost, transportation cost, and fixed cost of equipment to build a closed-loop supply chain network model, including a collection center and a remanufacturing center. The objective of the proposed model aimed to optimize the product flow along the network. Lundin and Johan [16] proposed a paper currency closed-loop supply chain network model and explored the impact of network planning when the risks, such as disruptions in the network structure (e.g. reduction in the number of storage facilities), business processes (e.g. outsourcing), and incentive mechanisms (e.g. payment schemes and policies), change. Özceylan et al. [17] presented a closed-loop supply chain linear programming model for the recovery of end-of-life vehicles in Turkey. Reza and Nasim [18] developed a mixed integer linear programming method based on quantity discounts for green logistics network configuration, inventory management decisions, and control of CO_2_ emissions. Hasanov et al. [19] discussed the coordination of order quantity and remanufacturing in a four-tiered closed-loop supply chain. The authors constructed a closed-loop supply chain planning model to design the optimization strategy of manufacturing, remanufacturing, and inventory to minimize the total cost of the closed-loop supply chain. Many other research efforts on closed-loop supply chains have been conducted over the last years focusing on different types of products, including tires, bread, glass, computer devices, batteries, mobile devices, and others [20]. Nevertheless, the aforementioned research efforts are mostly inclined toward the relevant studies of the closed-loop supply chain of a specific product or industry rather than taking into account the general closed-loop supply chain network design features.

Furthermore, there are many uncertain factors, which can easily cause major changes in the network structure, thus increasing the risk of profit loss for enterprises. Therefore, it is necessary to consider various sources of uncertainty in the closed-loop supply chain network design from a strategic perspective [4]. Some researchers [3, 21] used stochastic programming to investigate the uncertainty of the supply chain network. Given the complexity of the production and operation processes or the transience of the product life cycle, it is difficult to obtain the historical data required by stochastic programming [13]. Given this deficiency, researchers started using fuzzy theory, which has the advantage of not relying on historical data to deal with the uncertainty of certain parameters associated with closed-loop supply chain planning. As an example, Tofighi et al. [22] developed a fuzzy humanitarian closed-loop supply chain planning model by applying the fuzzy ranking method and the possibility planning method of credibility measurement. Govindan et al. [23] constructed a fuzzy closed-loop supply chain programming model, considering the recycling of inkjet printers and supplier selection. Soleimani et al. [24] established a sustainable fuzzy green closed-loop supply chain programming model that explicitly captured the product recycling processes. Chen et al. [25] suggested a closed-loop supply chain model with a new fuzzy controller embedded with quality indicators, considering the structural design and optimization of the product renovation process and the uncertainty. Ghomi-Avili et al. [26] proposed a fuzzy closed-loop supply chain programming model, considering supplier random interruptions and environmental issues. Ghahremani et al. [27] built a robust fuzzy location/distribution planning model, taking into account potential raw material procurement shortages, uncertainty, and discounts. Asim et al. [28] established a fuzzy closed-loop supply chain goal programming model, which integrated production and transportation, to minimize the total cost, total defects, and total delivery time. Thus, this study aims to develops a fuzzy programming model for the general closed-loop supply chain network by using the credibility measure-based fuzzy ranking method, which has the advantage of not depending on historical data for modeling uncertain parameters.

As a combinatorial optimization problem with NP-hard complexity, the design of a closed-loop supply chain network still remains a challenge, and there is a need for exploring more effective solution approaches [29, 30]. Migrating Birds Optimization (MBO) is a new meta-heuristic algorithm, which can reduce energy loss by simulating the “V” formation during migration of birds. It has the characteristics of few parameters, simple structure, and strong global and local search ability [31]. Duman et al. [31] first proposed the MBO algorithm in 2012 and successfully applied it to the secondary allocation problem. Since then, the algorithm has been used to solve flow shop [32, 33], traveling salesman [34], and flexible manufacturing systems [35]. However, there is a lack of studies on the application of the MBO algorithm for closed-loop supply chain network design.

The encoding method of the metaheuristic algorithm will affect the efficiency of the algorithm. For the closed-loop supply chain network design problem, most of the current literature mainly adopts the following encoding methods: Prüfer number-based encoding [36], spanning tree-based encoding [37], forest data structure-based encoding [38, 39], priority-based encoding [5, 40–47], to name a few. However, there are certain drawbacks associated with the aforementioned encoding methods. In particular, for some special problems with high computational complexity, these encoding methods do not allow the algorithms reaching the optimal solutions even for small-scale problem instances. In order to address this challenge, this study proposes a new encoding method that is mainly based on the product source coding.

In summary, this paper aims to construct a generalized strategic planning model for a multi-level closed-loop logistics network aiming to minimize the total cost of the associated supply chain operations. Uncertainty in various factors, such as demand, facility opening cost, transportation cost, facility processing cost, recovery rate, and remanufacturing rate, are directly incorporated within the proposed modelling framework [28]. The fuzzy ranking method based on the credibility measure is applied in order to address uncertainty of the aforementioned supply chain parameters [27]. Last by not least, a Migrating Birds Optimization Algorithm, which deploys a Product Source Coding (PSMBO) scheme for encoding the candidate solutions, is developed as a solution approach. The following sections of the manuscript elaborate more on the problem description and uncertainty modeling aspects. After that, the optimization model with fuzzy parameters is formally introduced along with its defuzzified formulation. Next, the manuscript provides a detailed review of the proposed solution approach and presents a set of computational examples to demonstrate the performance of the proposed methodology. The last section summarizes the present research efforts and provides some directions for the future research.

## Section 3: Problem description and uncertainty modeling

### 3.1. Problem definition

**Fig 1:**
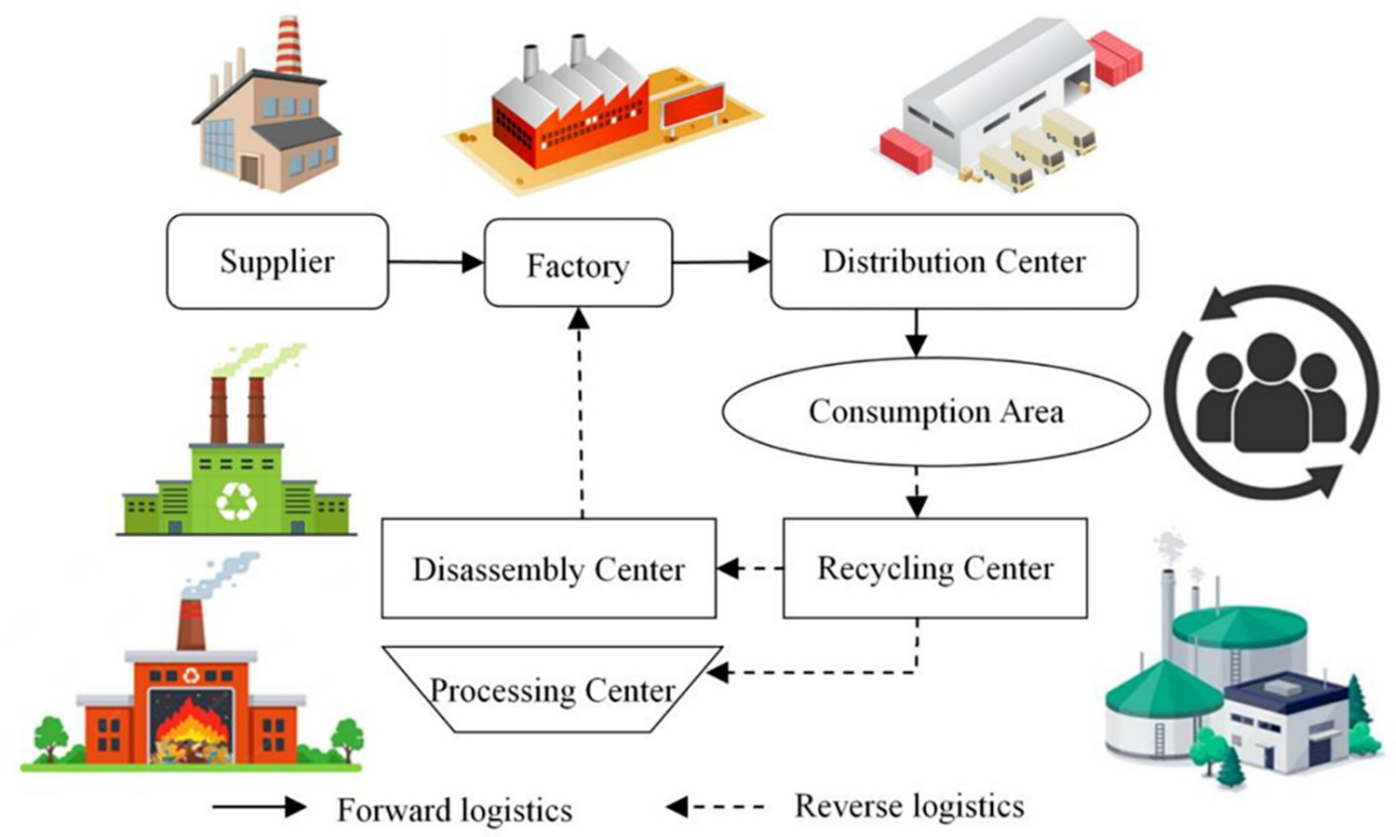
Closed-loop supply chain network structure.

This paper investigates a closed-loop supply chain network composed of suppliers, factories, distribution centers, consumption areas, recycling centers, disassembly centers, and processing centers (as shown in Fig 1). For the forward supply chain, the factories produces products. One portion of the raw materials used is purchased from the suppliers, and the other portion is provided by the disassembly centers. Then, the final products must be transported to the consumption area through the distribution center. In the reverse supply chain, the recycling center provides a certain compensation fee to consumers to obtain the used products and recycle the used products from the consumption area. Then, the recycling center also checks the quality of the used products and classifies them into recyclable products and non-recyclable products (i.e. the products that cannot be recycled anymore). Among them, the recyclable products are transported to the disassembly center and separated into different materials according to different operation methods, and then the recycled materials are transported to the factory and used in the factory for production. Non-recyclable products are transported to the processing centers. The closed-loop supply chain network model in this paper has a general structure, which can be used for recycling and processing of various types of products. It can be used in different types of industries, such as tire or glass manufacturing industries, with the appropriate modifications or without any modifications.

In the actual production operations, due to the complexity of the remanufacturing market, the uncertainty of factors, or the fuzziness of human thinking, it is difficult for enterprise managers to give an exact real value *b*^*M*^ for each supply chain parameter. However, *b*^*M*^ an approximate value of each parameter is typically known. Triangular fuzzy numbers (*b*^*L*^,*b*^*M*^,*b*^*R*^) have unique advantages when expressing a parameter value, and most managers are more willing to give the minimum (*b*^*L*^), most likely (*b*^*M*^), and maximum (*b*^*R*^) values of the evaluation scale. Hence, triangular fuzzy numbers are used to represent the uncertain parameters of the model. In summary, this study aims to formulate a decision problem of facility location and transportation route selection between the nodes in the closed-loop supply chain network to minimize the total cost, including the fixed cost of facility opening, operating facility cost, procurement cost, collection cost, and transportation cost. Moreover, the fuzzy programming method is adopted to deal with the uncertainty of the problem parameters and establish a fuzzy programming model for the closed-loop supply chain network.

### 3.2. Uncertainty modeling

Due to the intricate and dynamic nature of the market environment, the data acquired frequently exhibit uncertainty. For instance, factors such as the initial cost and capacity of facilities, transportation expenses influenced by labor availability and rates, fluctuations in freight charges, and demand impacted by product pricing and seasonal variations render these parameters inherently uncertain. As a result, they cannot be precisely quantified. To address these uncertainties, this paper posits that all uncertain values are represented as triangular fuzzy numbers. To tackle this issue, a closed-loop supply chain network model grounded in credibility theory within a fuzzy environment is formulated.

There are usually three types of mathematical models based on credibility measures [48]: expected value model [49], chance constrained programming model [50], and related chance programming model [51]. The expected value model is the simplest and the most convenient to use. It will not increase the complexity of the model and the speed of the computer, but it cannot give the confidence level of the chance constraint. The chance-constrained programming model gives the confidence level of the establishment of the chance-constrained programming model, and its confidence level directly or indirectly reflects the preference of the decision-maker and the size of the risk. The related chance programming model is similar to the chance constrained programming model, but it emphasizes the confidence level more, so it is more suitable for conservative decision makers. Through the above analysis, based on the credibility theory, combined with the expected value model and the chance-constrained programming model, the optimization model of the closed-loop supply chain network problem under the fuzzy environment is established.

### 3.3. Model assumptions and symbols

The following major assumptions were adopted in this study: (1) a single product type will be present in a single production cycle; (2) The number of facilities and their processing capabilities are limited; and (3) Each consumption area cooperates with the product recycling center and accepts the products manufactured from raw materials, which are provided by the supplier, and the products manufactured from recycled materials, which are provided by the disassembly center. The definition of symbols adopted in this study is provided in Table 3.

**Table 3:** Definition of mathematical symbols.

**Sets:**	
*I*: Set of fixed locations of suppliers, *i*∈*I*	*J*: Set of candidate factory locations, *j*∈*J*
*K*: Set of candidate distribution center locations, *k*∈*K*	*L*: Set of fixed locations of consumption areas, *l*∈*L*
*H*: Set of candidate recycling center locations, *h*∈*H*	*R*: Set of candidate disassembly center locations, *r*∈*R*
*M*: Set of candidate processing center locations, *m*∈*M*	*E*: Set of production processes, *e*∈*E*
**Parameters:**	
${\tilde{d}}_{l}$: Fuzzy demand of consumption area *l*	${\tilde{f}}_{j}^{1}$: Fuzzy fixed opening cost of factory *j*
${\tilde{f}}_{k}^{2}$: Fuzzy fixed opening cost of distribution center *k*	${\tilde{f}}_{h}^{3}$: Fuzzy fixed opening cost of recycling center *h*
${\tilde{f}}_{er}^{4}$: Fuzzy fixed opening cost of disassembly center *r*using production process *e*	${\tilde{f}}_{m}^{5}$: Fuzzy fixed opening cost of processing center *m*
${\tilde{t}}_{jk}^{1}$: Unit fuzzy transportation cost from factory *j* to distribution center *k*	${\tilde{t}}_{kl}^{2}$: Unit fuzzy transportation cost from distribution center *k* to consumption area *l*
${\tilde{t}}_{hr}^{3}$: Unit fuzzy transportation cost from recycling center *h* to disassembly center *r*	${\tilde{t}}_{rj}^{4}$: Unit fuzzy transportation cost from disassembly center *r* to factory *j*
${\tilde{t}}_{hm}^{5}$: Unit fuzzy transportation cost from recycling center *h* to processing center *m*	$p{\tilde{p}}_{i}$: Unit fuzzy cost of raw materials purchased from supplier *i*
$p{\tilde{o}}_{l}$: Unit fuzzy cost of purchasing used products from consumption area *l*	$m{\tilde{c}}_{j}$: Unit fuzzy cost of factory *j*manufactured products
$c{\tilde{c}}_{lh}$: The unit fuzzy cost of products collected from consumption area *l* to be used by recycling center *h*	$r{\tilde{c}}_{er}$: Unit fuzzy operation cost of disassembly center *r* performing production process *e*
${\tilde{c}}_{i}^{1}$: Fuzzy capacity of supplier *i*	${\tilde{c}}_{j}^{2}$: Fuzzy capacity of factory *j*
${\tilde{c}}_{k}^{3}$: Fuzzy capacity of distribution center *k*	${\tilde{c}}_{h}^{4}$: Fuzzy capacity of recycling center *h*
${\tilde{c}}_{er}^{5}$: Fuzzy capacity of disassembly center *r* performing production process *e*	${\tilde{c}}_{m}^{6}$: Fuzzy capacity of processing center *m*
${\tilde{\tau }}_{l}$: Fuzzy recovery rate of consumption area *l*	${\tilde{o}}^{r}$: Fuzzy remanufacturing rate
*Wh*: Weight per unit of non- remanufacturable product	
**Decision variables:**	
$X_{ij}^{1}$: Transportation volume from supplier *i* to factory *j*	$X_{jk}^{2}$: Transportation volume from factory *j* to distribution center *k*
$X_{kl}^{3}$: Transportation volume from distribution center *k* to consumption area *l*	$Y_{lh}^{1}$: Transportation volume from consumption area *l* to recycling center *h*
$Y_{ehr}^{2}$: Transportation volume from recycling center *h* to disassembly center *r* performing production process *e*	*U*_*erj*_: Transportation volume from disassembly center *r* using production process *e* to factory *j*
*V*_*hm*_: Transportation volume from recycling center *h* to processing center *m*	$B_{j}^{1}$: 1 when candidate factory *j* is selected, 0 otherwise
$B_{k}^{2}$: 1 when candidate distribution center *k* is selected, 0 otherwise	$B_{h}^{3}$: 1 when candidate recycling center *h* is selected, 0 otherwise
$B_{er}^{4}$: 1 when disassembly center *r* performing production process *e* is selected, 0 otherwise	$B_{m}^{5}$: 1 when candidate processing center *m* is selected, 0 otherwise
$\bar{f}$: Upper limit of the total cost	

### 3.4. Objective function and constraints of the fuzzy optimization model

For the fuzzy parameters of the closed-loop supply chain, this study uses the chance- constrained programming method to establish a fuzzy programming model [25] based on the fuzzy ranking method with credibility measures. The goal is to minimize the upper limit value of the total cost, where the total cost (*TC*) includes the fixed cost of opening the facilities (*FC*), the operating cost of the facilities (*MRC*), the procurement cost (*PC*), the collection cost (*CC*), and the transportation cost (*RC*). More specifically, *FC* represents the total opening cost of certain network facilities (i.e. factories, distribution centers, recycling centers, disassembly centers, and processing centers), *MRC* represents the total operating cost of processing products by the network facilities, *PC* represents the sum of the costs of purchasing raw materials and acquiring used products, *CC* represents the total cost of collecting used products, and *RC* represents the total transportation cost of raw materials or products between facilities,. The total cost of supply chain operations can be estimated as follows:

TC=FC+MRC+PC+CC+RC

Wherein:

$$FC=\sum_{j}{\tilde{f}}_{j}^{1}B_{j}^{1}+\sum_{k}{\tilde{f}}_{k}^{2}B_{k}^{2}+\sum_{h}{\tilde{f}}_{h}^{3}B_{h}^{3}+\sum_{e}\sum_{r}{\tilde{f}}_{er}^{4}B_{er}^{4}+\sum_{m}{\tilde{f}}_{m}^{5}B_{m}^{5}$$

$$MRC=\sum_{j}\sum_{k}m{\tilde{c}}_{j}X_{jk}^{2}+\sum_{e}\sum_{r}\sum_{j}r{\tilde{c}}_{er}U_{erj}$$

$$PC=\sum_{i}\sum_{j}p{\tilde{p}}_{i}X_{ij}^{1}+\sum_{l}\sum_{h}p{\tilde{o}}_{l}Y_{lh}^{1}$$

$$CC=\sum_{l}\sum_{h}c{\tilde{c}}_{lh}Y_{lh}^{1}$$

$$RC=\sum_{j}\sum_{k}{\tilde{t}}_{jk}^{1}X_{jk}^{2}+\sum_{k}\sum_{l}{\tilde{t}}_{kl}^{2}X_{kl}^{3}+\sum_{e}\sum_{h}\sum_{r}{\tilde{t}}_{hr}^{3}Y_{ehr}^{2}+\sum_{e}\sum_{r}\sum_{j}{\tilde{t}}_{rj}^{4}U_{erj}+\sum_{h}\sum_{m}{\tilde{t}}_{hm}^{5}V_{hm}$$

Therefore, the credibility-based fuzzy chance-constrained programming model M1 can be formulated as follows:

$$\min\bar{f}$$ (1)

According to the assumptions adopted in this study, the decision variables must meet the following constraints:

$$\mathrm{Cr}(TC\leq \bar{f})\geq \alpha$$ (2)

$$\mathrm{Cr}(\sum_{k}X_{kl}^{3}\geq {\tilde{d}}_{l})\geq \alpha ,\;\forall l\in L$$ (3)

$$\sum_{k}X_{jk}^{2}=\sum_{i}X_{ij}^{1}+\sum_{e}\sum_{r}U_{erj},\;\forall j\in J$$ (4)

$$\sum_{j}X_{jk}^{2}=\sum_{l}X_{kl}^{3},\;\forall k\in K$$ (5)

$$\mathrm{Cr}(\sum_{h}Y_{lh}^{1}=\lfloor {\tilde{\tau }}_{l}\sum_{k}X_{kl}^{3}\rfloor )\geq \alpha ,\;\forall l\in L$$ (6)

$$\sum_{l}Y_{lh}^{1}=\sum_{e}\sum_{r}Y_{ehr}^{2}+\sum_{m}\frac{V_{hm}}{Wh},\;\forall h\in H$$ (7)

$$\mathrm{Cr}(\sum_{e}\sum_{r}Y_{ehr}^{2}=\lceil \sum_{l}{\tilde{o}}^{r}Y_{lh}^{1}\rceil )\geq \alpha ,\;\forall h\in H$$ (8)

$$\sum_{h}Y_{ehr}^{2}=\sum_{j}U_{erj},\;\forall e\in E,r\in R$$ (9)

$$\mathrm{Cr}(\sum_{j}X_{ij}^{1}\leq {\tilde{c}}_{i}^{1})\geq \alpha ,\;\forall i\in I$$ (10)

$$\mathrm{Cr}(\sum_{k}X_{jk}^{2}\leq {\tilde{c}}_{j}^{2}B_{j}^{1})\geq \alpha ,\;\forall j\in J$$ (11)

$$\mathrm{Cr}(\sum_{l}X_{kl}^{3}\leq {\tilde{c}}_{k}^{3}B_{k}^{2})\geq \alpha ,\;\forall k\in K$$ (12)

$$\mathrm{Cr}(\sum_{l}Y_{lh}^{1}\leq {\tilde{c}}_{h}^{4}B_{h}^{3})\geq \alpha ,\;\forall h\in H$$ (13)

$$\mathrm{Cr}(\sum_{j}U_{erj}\leq {\tilde{c}}_{er}^{5}B_{er}^{4})\geq \alpha ,\;\forall e\in E,r\in R$$ (14)

$$\mathrm{Cr}(\sum_{h}V_{hm}\leq {\tilde{c}}_{m}^{6}B_{m}^{5})\geq \alpha ,\;\forall m\in M$$ (15)

$$\sum_{e}B_{er}^{4}\leq 1,\;\forall r\in R$$ (16)

$$\bar{f}\geq 0,\;\forall i,j,l,h,r,m,e$$ (17)

$$X_{ij}^{1},X_{jk}^{2},X_{kl}^{3},Y_{lh}^{1},Y_{ehr}^{2},U_{erj},V_{hm}\in \{0\}\cup N^{+},\;\forall i,j,k,l,h,r,m,e$$ (18)

$$B_{j}^{1},B_{k}^{2},B_{h}^{3},B_{er}^{4},B_{m}^{5}\in \{0,1\},\;\forall j,k,h,r,m,e$$ (19)

Note: ⌊ ⌋ indicates rounding down, ⌈ ⌉ indicates rounding up.

Wherein: Objective function (1) minimizes the upper limit value of the total cost. Constraint set (2) indicates that the total cost (*TC*) value cannot be higher than the upper limit value of the objective function, and its credibility cannot be less than *α*∈[0,1]. Constraint set (3) shows that for the corresponding consumers, the reliability of the number of products transported to consumers by the distribution center to meet the needs of consumers cannot be less than *α*∈[0,1]. Constraint sets (4) to (9) are the flow balance constraints, indicating that the input of the facility is equal to the output, where constraint set (6) represents the quantity of used products transported from the consumption area to the recycling center. The integer value of the recycling volume is rounded down according to the fuzzy recovery rate and the total quantity of products transported from the distribution center to the consumption area, and its credibility cannot be less than *α*∈[0,1]. Similarly, the credibility of constraint set (8) cannot be less than *α*∈[0,1], when estimating the volume of used products transported from the recycling center to the disassembly center. Constraint sets (10) to (15) indicate that the capacity of each facility would be sufficient to handle raw materials or products, with the credibility not less than *α*∈[0,1]. Constraint set (16) indicates that each disassembly center can only select one operation mode at the most. Constraint set (17) ensures that the upper limit on the total objective function is non-negative. Constraint sets (18) and (19) are the cardinality constraints for the decision variables of the presented mathematical model.

Since $X_{kl}^{3}$ is a decision variable and $\tau_{l}\sum_{k\in K}X_{kl}^{3}$ cannot be rounded directly, constraint set (6) can be adjusted as follows:

$$\mathrm{Cr}(\sum_{h}Y_{lh}^{1}\leq {\tilde{\tau }}_{l}\sum_{k}X_{kl}^{3})\geq \alpha ,\;\forall l\in L$$ (20)

$$\mathrm{Cr}(\sum_{h}Y_{lh}^{1}\geq {\tilde{\tau }}_{l}\sum_{k}X_{kl}^{3}-\varepsilon )\geq \alpha ,\;\forall l\in L$$ (21)

Wherein, *ε*→1^−^ ∀*i*,*j*,*j*,*l*,*h*,*r*,*m*,*c*,*e*, Constraint sets (20) and (21) represent the largest integer limiting $\sum_{h}Y_{lh}^{1}$ to no more than ${\tilde{\tau }}_{l}\sum_{h}X_{kl}^{3}$.

Similarly, $Y_{lh}^{1}$ is a decision variable and $\sum_{l}o^{r}Y_{lh}^{1}$ cannot be rounded directly. Therefore, constraint set (8) can be adjusted as follows:

$$\mathrm{Cr}(\sum_{e}\sum_{r}Y_{ehr}^{2}\geq \sum_{l}{\tilde{o}}^{r}Y_{lh}^{1})\geq \alpha ,\;\forall h\in H$$ (22)

$$\mathrm{Cr}(\sum_{e}\sum_{r}Y_{ehr}^{2}\leq \sum_{l}{\tilde{o}}^{r}Y_{lh}^{1}+\varepsilon )\geq \alpha ,\;\forall h\in H$$ (23)

Wherein, constraint sets (22) and (23) represent the minimum integer limiting $\sum_{e}\sum_{r}Y_{ehr}^{2}$ to greater than $\sum_{l}{\tilde{o}}^{r}Y_{lh}^{1}$.

### 3.5. Defuzzified model formulation

Based on the analysis of the available literature [23, 24], this paper adopts a new fuzzy ranking method based on credibility measures to deal with the chance constraints in the optimization model and address uncertainty in the values of the model parameters. Let $\tilde{a}$ and $\tilde{b}$ be two possibilistic parameters represented by the following triangular probability distributions (i.e. two fuzzy numbers):

$\tilde{a}$ and $\tilde{b}$ are triangular fuzzy numbers: $\tilde{a}=TFN(a_{1},a_{2},a_{3})$, $\tilde{b}=TFN(b_{1},b_{2},b_{3})$ The credibility measure $\tilde{a}\leq \tilde{b}$ can be defined as follows [22]:

$$\mathrm{Cr}(\tilde{a}\leq \tilde{b})=\frac{1}{2}(\mathrm{Pos}(\tilde{a}\leq \tilde{b})+\mathrm{Nec}(\tilde{a}\leq \tilde{b}))$$ (24)

wherein, the probability degree $\tilde{a}\leq \tilde{b}$ can be expressed using the following relationship:

$$\mathrm{Pos}(\tilde{a}\leq \tilde{b})=\{\begin{array}{l} 0,\;a_{1}\geq b_{3}\\ \frac{b_{3}-a_{1}}{b_{3}-b_{2}+a_{2}-a_{1}},a_{2}\geq b_{2},a_{1}\leq b_{3}\\ 1,\;a_{2}\leq b_{2} \end{array}$$

The necessity measure $\tilde{a}\leq \tilde{b}$ can be defined as: $Nec(\tilde{a}\leq \tilde{b})=1-Pos(\tilde{a}\leq \tilde{b})$ Now, the fuzzy ranking Formula (24) can be reformulated according to the reliability measurement:

$$\mathrm{Cr}(\tilde{a}\leq \tilde{b})=\{\begin{array}{l} 0,\;a_{1}\geq b_{3}\\ \frac{b_{3}-a_{1}}{2(b_{3}-b_{2}+a_{2}-a_{1})},a_{2}\geq b_{2},a_{1}\leq b_{3}\\ \frac{a_{3}-b_{1}+2b_{2}-2a_{2}}{2(b_{2}-b_{1}+a_{3}-a_{2})},a_{2}\leq b_{2},a_{3}\geq b_{1}\\ 1,\;a_{2}\leq b_{2} \end{array}$$

Therefore, the credibility measure of $\tilde{a}\leq \tilde{b}$ at the confidence level *α* can be defined as follows:

$$\mathrm{Cr}(\tilde{a}\leq \tilde{b})\geq \alpha \equiv \{\begin{array}{l} \frac{b_{3}-a_{1}}{2(b_{3}-b_{2}+a_{2}-a_{1})}\geq \alpha ,0\leq \alpha \leq 0.5\\ \frac{a_{3}-b_{1}+2b_{2}-2a_{2}}{2(b_{2}-b_{1}+a_{3}-a_{2})}\geq \alpha ,0.5\leq \alpha \leq 1 \end{array}$$

Where $\tilde{a}$ and $\tilde{b}$ are triangular fuzzy numbers.

The clear equivalent class of fuzzy inequalities $Cr(\tilde{a}\leq \tilde{b})\geq \alpha$ can be expressed in a simplified form as follows:

$$a^{c}+(2\alpha -1)w_{a}\leq b^{c}+(1-2\alpha )w_{b},\;\forall \alpha \in [0,1]$$

wherein, *a*^*c*^ and *w*_*a*_ respectively represent the midpoint and extension range of symmetric fuzzy numbers related to $\tilde{a}$; similarly, *b*^*c*^ and *w*_*b*_ respectively represent the midpoint and extension range of symmetric fuzzy numbers related to $\tilde{b}$.

If all fuzzy parameters are simply regarded as symmetric triangular fuzzy numbers, both sides having a range of 20%, that is $\tilde{a}=\langle a^{c},0.2a^{c}\rangle \equiv \mathrm{TFN}(0.8a^{c},a^{c},1.2a^{c})$, then the following relationship will be valid:

$$\left(0.8+0.4\alpha )a^{c}\leq (1.2-0.4\alpha )b^{c},\;\forall \alpha \in [0,1\right]$$ (25)

Since the optimization model M1 with the fuzzy chance constraint sets is difficult to be solved directly with a commercial solver, the transformation process from Eq (24) to Eq (25) is used to change the fuzzy chance constraints in model M1 into defuzzified equivalent constraint sets as follows:

$$\begin{array}{l} \sum_{j}f_{j}^{1c}(0.8+0.4\alpha )B_{j}^{1}+\sum_{k}f_{k}^{2c}(0.8+0.4\alpha )B_{k}^{2}+\sum_{h}f_{h}^{3c}(0.8+0.4\alpha )B_{h}^{3}\\ +\sum_{e}\sum_{r}f_{er}^{4c}(0.8+0.4\alpha )B_{er}^{4}+\sum_{m}f_{m}^{5c}(0.8+0.4\alpha )B_{m}^{5}+\sum_{j}\sum_{k}mc_{j}^{c}(0.8+0.4\alpha )X_{jk}^{2}\\ +\sum_{e}\sum_{r}\sum_{j}rc_{er}^{c}(0.8+0.4\alpha )U_{erj}+\sum_{i}\sum_{j}pp_{i}^{c}(0.8+0.4\alpha )X_{ij}^{1}+\sum_{l}\sum_{h}po_{l}^{c}(0.8+0.4\alpha )Y_{lh}^{1}\\ +\sum_{l}\sum_{h}cc_{lh}^{c}(0.8+0.4\alpha )Y_{lh}^{1}+\sum_{j}\sum_{k}t_{jk}^{1c}(0.8+0.4\alpha )X_{jk}^{2}+\sum_{k}\sum_{l}t_{kl}^{2c}(0.8+0.4\alpha )X_{kl}^{3}\\ +\sum_{e}\sum_{h}\sum_{r}t_{hr}^{3c}(0.8+0.4\alpha )Y_{ehr}^{2}+\sum_{e}\sum_{r}\sum_{j}t_{rj}^{4c}(0.8+0.4\alpha )U_{erj}+\sum_{h}\sum_{m}t_{hm}^{5c}(0.8+0.4\alpha )V_{hm}\leq \bar{f} \end{array}$$ (26)

$$\sum_{k}X_{kl}^{3}\geq d_{l}^{c}(0.8+0.4\alpha ),\;\forall l\in L$$ (27)

$$\sum_{j}X_{ij}^{1}\leq c_{i}^{1c}(1.2-0.4\alpha ),\;\forall i\in I$$ (28)

$$\sum_{k}X_{jk}^{2}\leq c_{j}^{2c}(1.2-0.4\alpha )B_{j}^{1},\;\forall j\in J$$ (29)

$$\sum_{l}X_{kl}^{3}\leq c_{k}^{3c}(1.2-0.4\alpha )B_{k}^{2},\;\forall k\in K$$ (30)

$$\sum_{l}Y_{lh}^{1}\leq c_{h}^{4c}(1.2-0.4\alpha )B_{h}^{3},\;\forall h\in H$$ (31)

$$\sum_{j}U_{erj}\leq c_{er}^{5c}(1.2-0.4\alpha )B_{er}^{4},\;\forall e\in E,r\in R$$ (32)

$$\sum_{h}V_{hm}\leq c_{m}^{6c}(1.2-0.4\alpha )B_{m}^{5},\;\forall m\in M$$ (33)

$$\sum_{h}Y_{lh}^{1}\leq \tau_{l}^{c}(1.2-0.2\alpha )\sum_{k}X_{kl}^{3},\;\forall l\in L$$ (34)

$$\sum_{h}Y_{lh}^{1}\geq \tau_{l}^{c}(0.8+0.2\alpha )\sum_{k}X_{kl}^{3}-\varepsilon ,\;\forall l\in L$$ (35)

$$\sum_{e}\sum_{r}Y_{ehr}^{2}\geq \sum_{l}o^{rc}(0.8+0.2\alpha )Y_{lh}^{1},\;\forall h\in H$$ (36)

$$\sum_{e}\sum_{r}Y_{ehr}^{2}\leq \sum_{l}o^{rc}(1.2-0.2\alpha )Y_{lh}^{1}+\varepsilon ,\;\forall h\in H$$ (37)

Therefore, this study establishes a closed-loop supply chain network fuzzy programming model M2 with Eq (1) as the objective function and constraint sets (4), (5), (7), (9), (16) to (19), and (26) to (37).

## Section 4: Solution method

The Migratory Birds Optimization Algorithm (MBO) simulates the pressure formed by the flight of migratory birds, which helps the rear migratory birds rise to save their energy consumption, thus improving the flight distance of migratory birds. The MBO algorithm starts from a parallel solution, and the special search mode makes the algorithm expanding the exploration of the search space, showing the decentralized search ability of the algorithm. In addition, individuals in the flock can find better solutions by searching their neighborhood solutions and the neighborhood solutions of the individuals in front of them, which shows the centralized search ability of the algorithm. Therefore, the MBO algorithm possesses both global and local search capabilities and has a strong optimization ability. By taking the advantage of global and local search capabilities of MBO, this study proposes a Migrating Birds Optimization Algorithms with Product Source Coding (PSMBO) to solve the model developed for a closed-loop supply chain network.

### 4.1 Encoding and decoding operation based on the product source

The encoding method based on the product source proposed in this study refers to the unit product starting from a certain node and delivered to the consumption area after passing through some intermediate nodes. For certain value *α* of the model M2 in this paper, the values of all the decision variables can be determined based on the proposed encoding scheme. The specific steps for encoding based on the product source are as follows:

**Step 1** Initialize the data: *I* is the set of fixed locations of suppliers, *i*∈*I*; *J* is the set of candidate factory locations, *j*∈*J*; *K* is the set of candidate distribution center locations, *k*∈*K*; *L* is the set of the fixed locations of the consumption areas, *l*∈*L*; *H* is the set of candidate recycling center locations, *h*∈*H*; *R* is the set of candidate disassembly center locations, *r*∈*R*; *M* is the set of candidate processing center locations, *m*∈*M*; *E* is the set of production processes, *e*∈*E*; *d*_1_ is the demand of the consumption area; $c_{i}^{1}$ is the capacity of supplier *i*; $c_{j}^{2}$ is the capacity of factory *j*; $c_{k}^{3}$ is the capacity of distribution center *k*; $c_{h}^{4}$ is the capacity of recycling center *h*; $c_{er}^{5}$ is the capacity of disassembly center *r* for production process *e*; $c_{m}^{6}$ is the capacity of processing center *m*; *τ*_1_ is the recovery rate of consumption area *l*; *o*^*r*^ is the remanufacturing rate.

**Step 2** Create an empty matrix **Z** with the required number of rows and 9 columns. For example, if three consumption areas need 2 units of products each, the empty matrix **Z** will be initialized as a 6×9 matrix, and the first row represents the nodes. The empty **Z** matrix can be expressed as:

**Table:** 

*L*	*H*	*M*	*R*	*E*	*I*	*J*	*K*	*L*

**Step 3** Fill in the last column of empty matrix **Z** after random sorting according to index *l* contained in *d*_*l*_. According to the data adopted in the above steps, there are two indexes of “1”, two indexes of “2”, and two indexes of “3” randomly sorted as [3 1 2 1 3 2] and filled in matrix **Z**:

**Table:** 

*L*	*H*	*M*	*R*	*E*	*I*	*J*	*K*	*L*
								3
								1
								2
								1
								3
								2

**Step 4** Randomly sort according to index *j* contained in $c_{j}^{2}$ and index *k* contained in $c_{k}^{3}$, and fill the first $\sum_{l}d_{l}$ numbers into the third last, and second last columns of the empty matrix **Z**, respectively. According to the data adopted in the above steps, if there are two factories (*J*) and four distribution centers (*K*), for example, take the first six numbers, which are randomly sorted as [2 2 1 1 2 1] and [4 3 2 4 2 3], respectively, and fill them in the third last and second last columns of matrix **Z**:

**Table:** 

*L*	*H*	*M*	*R*	*E*	*I*	*J*	*K*	*L*
						2	4	3
						2	3	1
						1	2	2
						1	4	1
						2	2	3
						1	3	2

**Step 5** Fill in the first column of empty matrix **Z** after random sorting according to index *l* contained in the product of the recovery rate *τ*_*l*_ and *d*_*l*_, and fill the first $\left\lceil \sum_{l}\tau_{l}d_{l}\right\rceil$ numbers into the second column of empty matrix **Z** after random sorting according to index $c_{h}^{4}$ contained *h*. According to the data adopted in the above steps, suppose there are two recycling centers (*H*), and the recovery rate is 50%, then three numbers are taken (i.e. 6 * 50% = 3 numbers), which are randomly sorted as [2 1 3] and [1 1 2], and filled into the first and second columns of matrix **Z**, respectively:

**Table:** 

*L*	*H*	*M*	*R*	*E*	*I*	*J*	*K*	*L*
2	1					2	4	3
1	1					2	3	1
3	2					1	2	2
0	0					1	4	1
0	0					2	2	3
0	0					1	3	2

**Step 6** Randomly sort according to the indexes of *r* and *e* contained in $c_{er}^{5}$ and fill the first $\left\lceil \sum_{r}o^{r}\sum_{l}\tau_{l}d_{l}\right\rceil$ numbers into the fourth and fifth columns of empty matrix **Z**, and randomly sort according to the index of *m* contained in $c_{m}^{6}$ and fill the first $\left(\left\lceil \sum_{l}\tau_{l}d_{l}\rceil -\lceil \sum_{r}o^{r}\sum_{l}\tau_{l}d_{l}\right\rceil \right)$ numbers into the third column of empty matrix **Z**. According to the data adopted in the above steps, assuming that the recycling rate is 60%, there are three recycling centers (*R*), three processing centers (*M*) and two production processes (*E*), which are randomly sorted as [1], [2 1] and [3 1], and filled in the third, fourth and fifth columns of matrix **Z**, respectively:

**Table:** 

*L*	*H*	*M*	*R*	*E*	*I*	*J*	*K*	*L*
2	1	0	2	3		2	4	3
1	1	0	1	1		2	3	1
3	2	1	0	0		1	2	2
0	0	0	0	0		1	4	1
0	0	0	0	0		2	2	3
0	0	0	0	0		1	3	2

**Step 7** Randomly sort according to the index of *i* contained in $c_{i}^{1}$ and fill the last $\left(\sum_{l}d_{l}-\lceil \sum_{r}o^{r}\sum_{l}\tau_{l}d_{l}\rceil \right)$ numbers into the sixth column of the empty matrix **Z** to obtain a complete nonempty matrix **Z**. According to the data adopted in the above steps, suppose there are three suppliers (*I*), and fill the last four randomly generated numbers [2 2 3 1] into the sixth column of matrix **Z**, as follows:

**Table:** 

*L*	*H*	*M*	*R*	*E*	*I*	*J*	*K*	*L*
2	1	0	2	3	0	2	4	3
1	1	0	1	1	0	2	3	1
3	2	1	0	0	2	1	2	2
0	0	0	0	0	2	1	4	1
0	0	0	0	0	3	2	2	3
0	0	0	0	0	1	1	3	2

After the above 7 steps are completed, a feasible solution can be generated and can represent an individual of the population of migrating birds. The decoding operation is as follows: according to the matrix **Z** obtained in step 7, the number codes between nodes are accumulated for product volumes. For example, according to the first two columns of the first row of the table, $Y_{21}^{1}$ = 1 can be obtained, and the values $X_{ij}^{1},X_{jk}^{2},X_{kl}^{3},Y_{lh}^{1},Y_{ehr}^{2},U_{erj},V_{hm}$ can be obtained in a similar fashion.

### 4.2. Population initialization

Assuming that the population size is *N*, this study uses the encoding operation based on the product source described in Section 5.1 to generate N individuals. After decoding, the left part of Eq (26) is used to calculate the objective function, and take the individual with the lowest objective function value (i.e. the fittest individual) as the leader bird. Then, the encoding operation based on the product source described in Section 5.1 is used again to generate (*N*-1) individuals. After decoding the (*N*-1) individuals, the left part of Eq (26) is used to calculate the fitness values of these individuals. The obtained values are arranged from the smallest to the largest. The individuals with even numbers in the population are placed in the left queue *L*, and the individuals with odd numbers in the population are placed in the right queue *R*, forming the left and right rows of birds in the *V* formation.

### 4.3. Neighborhood search

The individuals of PSMBO seek the promising locations of the search space through their neighborhood search and the neighborhood solutions of the immediately preceding individuals. Therefore, the efficiency of neighborhood search is crucial to the performance of the algorithm. In this paper, we designed the following two neighborhood search methods, *NS*_*1*_ and *NS*_*2*_:

1. NS_1_: Two non-zero digits in a column of random matrix **Z** are exchanged. Take matrix **Z** obtained in Section 5.1 as an example, and the operation results can be presented as follows (i.e. two integer values in the second column of matrix **Z** have been updated):
2. NS_2_: A non-zero number in a column of random matrix **Z** is randomly changed. Take matrix **Z** obtained in Section 5.1 as an example, and the operation results can be presented as follows (i.e. one integer value in the eighth column of matrix **Z** has been updated):

**Table:** 

*L*	*H*	*M*	*R*	*E*	*I*	*J*	*K*	*L*
2	**2**	0	2	3	0	2	4	3
1	1	0	1	1	0	2	3	1
3	**1**	1	0	0	2	1	2	2
0	0	0	0	0	2	1	4	1
0	0	0	0	0	3	2	2	3
0	0	0	0	0	1	1	3	2

**Table:** 

*L*	*H*	*M*	*R*	*E*	*I*	*J*	*K*	*L*
2	1	0	2	3	0	2	4	3
1	1	0	1	1	0	2	3	1
3	2	1	0	0	2	1	2	2
0	0	0	0	0	2	1	4	1
0	0	0	0	0	3	2	**3**	3
0	0	0	0	0	1	1	3	2

### 4.4. Main steps of PSMBO

**Step 1**: Initialize. Set the parameters and the maximum number of iterations of the algorithm, *Genmax*, so that the flag value is set to *f* = 1 and the iteration counter is set to *g* = 1. Initialize the population and form a *V*-shaped queue using the method described in Section 5.2.

**Step 2**: Lead the birds to evolve. According to the neighborhood search, *NS*_*1*_ and *NS*_*2*_ each generate *m*/2 neighborhood solutions of the leading bird. If the identified solution is better than the current leading bird, replace the leading bird with it, and add the unused *n* best neighborhood solutions to the shared sets *P*_*L*_ and *P*_*R*_.

**Step 3**: Evolve the population of birds. For each individual in the left queue *L*, search the neighborhoods using the *NS*_*1*_ and *NS*_*2*_ search methods to generate (*m*-*n*)/2 neighborhood solutions, respectively. If the best solution in the combination of (*m*-*n*) neighborhood solutions and P_L_ is better than the current solution, replace the current individual with that solution. Clear *P*_*L*_, and add (*m*-*n*) neighborhood solutions and *n* best solutions not used in the union set of *P*_*L*_ to *P*_*L*_. Conduct the same procedure for the right queue *R*.

**Step 4**: Determine whether the maximum number of rounds *T* has been reached. If not, go to step 2; otherwise, go to step 5.

**Step 5**: Update the current best solution, conduct a neighborhood search, and replace the worst solution in the current population with the new solution.

**Step 6**: Replace the leader bird. If *f* = 1, the first individual in *L* will be the new leader bird, move the leader bird to the end of *L* team, and set *f* = 0; Otherwise, the first individual in *R* will be the new leader bird, move the leader bird to the end of *R* team, and set *f* = 1.

**Step 7**: Make *g* = *g*+1 and judge whether *g*>*Genmax* is met. If yes, go to step 8; Otherwise, go to step 2.

**Fig 2:**
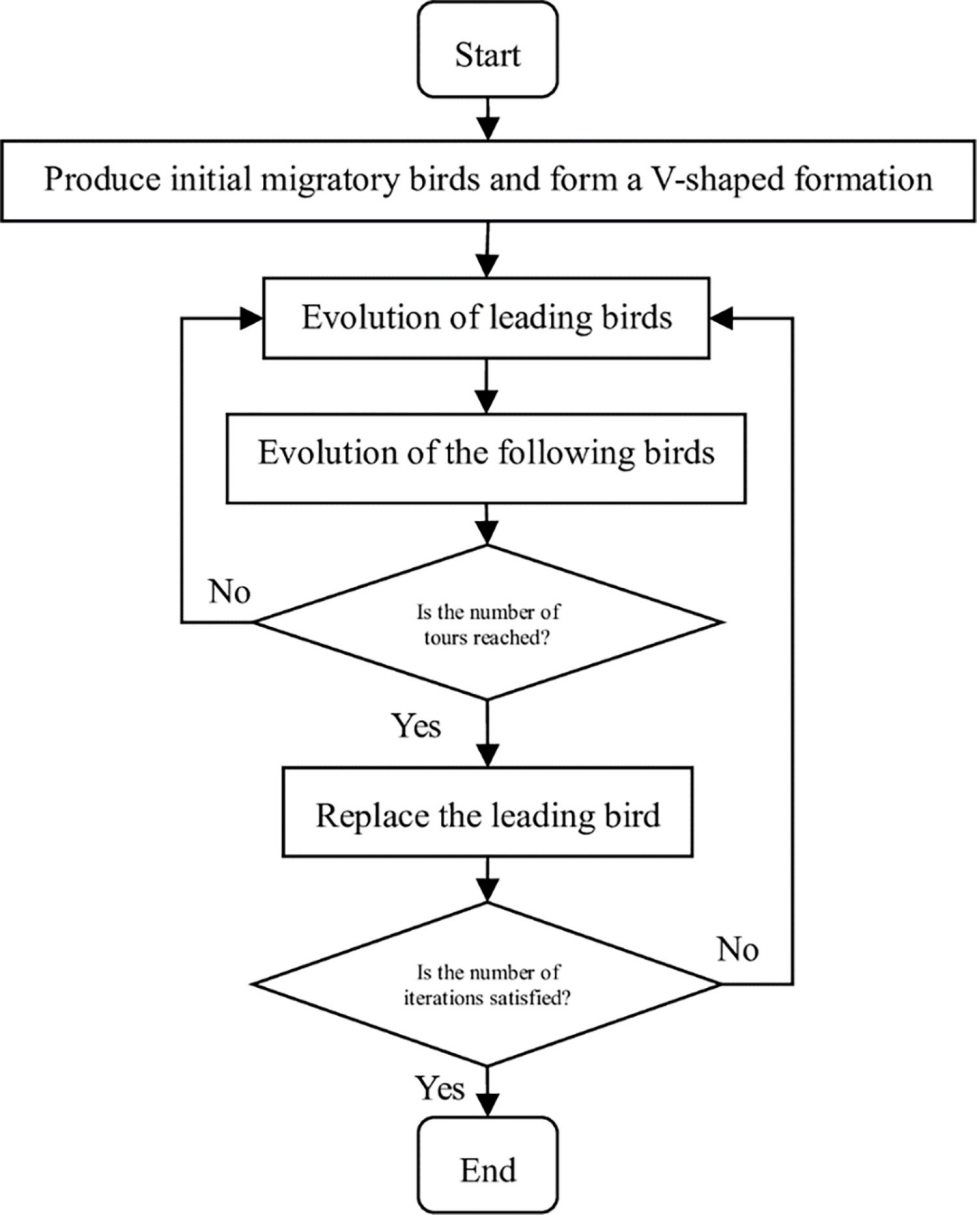
Solution flowchart of the migrating birds optimization algorithms based on product source coding.

**Step 8**: The algorithm is terminated, and the results can be retrieved. The outline of the main algorithmic steps is shown in Fig 2.

## Section 5: Numerical experiments

### 5.1. Input data generation

To evaluate the PSMBO algorithm, a Genetic Algorithm (GA), Ant Colony Optimization (ACO), Simulated Annealing (SA) and LINGO11 are were used for comparison. A detailed description of the GA, ACO and SA algorithm that were used in this study can be found in [32, 44]. The GA, ACO and SA were applied to the supply chain network model under an uncertain environment. The adopted GA, ACO and SA relied on the priority-based coding, which is similar to Prüfer number coding, spanning tree coding, forest data structure coding, etc. Similar to the PSMBO algorithm, GA, ACO and SA also have the characteristics of global search (by means of the crossover operations) and local search (by means of the mutation operations). Therefore, GA, ACO and SA would be an appropriate candidate solution approach that can be used for comparison with the PSMBO algorithm. The PSMBO, GA, ACO and SA algorithms were programmed using MATLAB7.0. The computational experiments were performed on a laptop with the Intel (R) Core (TM) i7 2.70GHz processor, 4GB RAM memory, and Windows 10 (64bit) operating system. The input data for numerical examples in this study were randomly generated from the values presented in Tables 4 and 5. The confidence level was set as 1. Based on the conducted parameter tuning analysis, the crossover and mutation rates of the GA algorithm were set to 60%~70% and 10%~15%, respectively.

**Table 4:** Characteristics of numerical examples considered in this study.

Example	Supplier	Factory	Distribution Center	Consumption area	Recycling Center	Disassembly center	Processing center	Production process
1	5	4	6	10	4	3	5	4
2	6	5	7	13	5	4	7	4
3	7	6	8	15	6	5	8	4
4	9	7	10	18	7	5	9	4
5	10	8	12	20	8	6	10	4
6	13	10	15	25	10	7	13	4
7	15	12	18	30	12	8	15	4
8	18	14	21	35	14	10	18	4
9	20	16	24	40	16	12	20	4
10	22	18	28	46	18	14	23	4
11	25	20	32	52	21	16	27	4
12	28	22	36	58	23	18	30	4
13	32	24	40	64	26	20	33	4
14	36	27	43	70	28	21	35	4
15	38	29	46	76	30	23	37	4
16	40	32	48	80	32	24	40	4
17	42	33	51	84	34	26	42	4
18	45	34	53	89	35	27	44	4
19	47	36	55	93	37	29	46	4
20	48	38	58	98	38	30	50	4
21	50	40	60	102	40	31	52	4
22	52	42	63	107	41	32	54	4
23	54	44	65	111	43	33	56	4
24	56	46	68	115	45	34	58	4
25	58	47	70	118	46	35	59	4
26	60	48	72	120	48	36	60	4
27	62	50	75	125	50	37	62	4
28	64	52	78	130	52	39	65	4
29	66	54	81	135	54	40	67	4
30	68	56	84	140	56	41	70	4
31	70	58	87	145	58	43	72	4
32	72	60	90	150	60	45	74	4
33	74	62	93	155	62	46	76	4
34	78	63	95	158	63	47	78	4
35	80	64	96	160	64	48	80	4

**Table 5:** Range of parameters in this study.

Parameters	Range	Parameters	Range
$d_{l}^{c}$	Uniform(10,30)	$f_{j}^{1c}$	Uniform(23000,24000)
$f_{k}^{2c}$	Uniform(12000,13000)	$f_{h}^{3c}$	Uniform(10000,12000)
$f_{er}^{4c}$	Uniform(10000,12000)	$f_{m}^{5c}$	Uniform(10000,13000)
$t_{jk}^{1c}$	Uniform(60,80)	$t_{kl}^{2c}$	Uniform(50,70)
$t_{hr}^{3c}$	Uniform(40,60)	$t_{rj}^{4c}$	Uniform(20,30)
$t_{hm}^{5c}$	Uniform(40,60)	$pp_{i}^{c}$	Uniform(55,60)
$po_{l}^{c}$	Uniform(200,240)	$mc_{j}^{c}$	Uniform(450,500)
$cc_{lh}^{c}$	Uniform(100,120)	$rc_{er}^{c}$	Uniform(30,35)
$c_{i}^{1c}$	Uniform(180,250)	$c_{j}^{2c}$	Uniform(200,320)
$c_{k}^{3c}$	Uniform(200,300)	$c_{h}^{4c}$	Uniform(225,290)
$c_{er}^{5c}$	Uniform(200,320)	$c_{m}^{6c}$	Uniform(150,280)
$\tau_{l}^{c}$	Uniform(0.58,0.83)	*o* ^*rc*^	Uniform(0.8,0.9)
*Wh*	Uniform(20,25)		

### 5.2. Evaluation of the candidate solution approaches

The PSMBO, GA, ACO and SA algorithms were run for 20 times for each example to obtain the average objective function and CPU time values. LINGO was executed for each example as well in order to identify the global optimal solution. The results of the performed analysis are summarized in **Table 6** (the bold number represents the optimal solution, and the mark "*" represents the feasible solution) and **Table 7**. As shown in **Table 6**, for examples, from 1 to 8, although the running time of PSMBO was significantly longer than that of LINGO, the target value of PSMBO was the same as that of LINGO, indicating that PSMBO can be used for small-scale problems, but its efficiency is lower than that of LINGO. For examples from 9 to 25, although the objective function value obtained by PSMBO is slightly larger than that of LINGO, the running time of PSMBO is much shorter than that of LINGO, which indicates that the precision of PSMBO can be viewed as acceptable, and its computational time is competitive. For example 26, the objective function value obtained by PSMBO is smaller than that of LINGO and its running time is shorter than that of LINGO. At the same time, for example, from 27 to 35, LINGO could not provide a feasible solution even due to insufficient system memory, while PSMBO was able to obtain solutions within an acceptable time range. Therefore, the numerical experiments clearly show advantages of PSMBO against the exact optimization method, especially for large-scale examples.

**Table 6:** Objective function values and CPU times of the considered solution approaches.

	LINGO		PSMBO			GA		
Example	Objective value	CPU time (seconds)	Objective value	Average value	CPU time (seconds)	Objective value	Average value	CPU time (seconds)
1	**271,386**	5.12	271,386	272,152.65	38.85	271,415	272,986.35	37.63
2	**352,498**	6.56	352,498	352,547.36	41.39	352,637	352,745.94	39.68
3	**451,636**	8.74	451,636	451,841.39	51.47	451,841	451,947.16	49.09
4	**505,711**	10.28	505,711	505,947.17	66.84	505,948	506,647.48	62.75
5	**541,762**	12.56	541,762	541,857.53	79.53	542,334	543,532.65	73.61
6	**722,356**	79.53	722,356	722,547.09	115.37	722,687	722,874.36	113.69
7	**902,950**	148.25	902,950	902,984.12	134.28	903,687	904,674.35	131.68
8	**993,247**	251.64	993,247	993,457.94	157.19	993,584	993,748.68	150.68
9	**1,083,544**	385.74	1,085,362	1,086,256.48	169.09	1,094,254	1,107,317.35	159.09
10	**1,264,634**	924.36	1,266,217	1,266,475.46	186.39	1,266,847	1,266,987.25	181.68
11	**1,445,725**	1549.38	1,449,274	1,449,348.31	205.36	1,449,984	1,450,159.39	201.67
12	**1,626,815**	2134.98	1,637,357	1,637,714.65	224.82	1,637,674	1,637,845.65	219.85
13	**1,807,906**	2527.36	1,818,395	1,818,579.16	246.39	1,818,589	1,818,745.34	239.68
14	**1,988,997**	3217.24	1,996,426	1,996,647.38	284.62	1,996,657	1,996,854.92	279.58
15	**2,079,542**	3557.31	2,098,049	2,098,247.62	303.49	2,098,374	2,098,547.69	297.68
16	**2,170,088**	4217.62	2,172,362	2,174,528.79	331.18	2,195,625	2,192,412.36	321.37
17	**2,333,762**	4841.08	2,334,247	2,334,514.08	351.42	2,343,574	2,343,684.25	348.68
18	**2,497,436**	5467.28	2,504,953	2,505,472.18	377.24	2,512,657	2,512,869.21	368.58
19	**2,661,110**	6146.37	2,684,305	2,684,516.37	393.65	2,695,748	2,695,984.58	386.69
20	**2,834,784**	6681.43	2,867,342	2,867,552.33	403.89	2,878,945	2,879,147.36	396.47
21	**2,988,459**	7276.14	2,995,219	2,995,473.68	416.26	2,999,247	2,999,567.49	409.39
22	**3,152,133**	8208.73	3,174,427	3,174,627.13	433.17	3,186,635	3,186,876.28	426.75
23	**3,315,807**	8731.92	3,335,164	3,335,376.49	456.89	3,343,175	3,343,348.24	445.63
24	**3,479,481**	9216.25	3,496,729	3,496,987.17	469.16	3,499,637	3,499,869.57	458.95
25	**3,544,951**	9637.19	3,556,049	3,556,371.27	483.75	3,565,540	3,565,769.18	475.93
26	3,643,156*	>9879.89	3,642,095	3,644,186.32	499.27	3,671,131	3,672,273.51	481.65
27	--	--	3,729,335	3,729,547.27	513.46	3,736,685	3,736,869.57	508.68
28	--	--	3,816,575	3,816,743.58	538.35	3,835,472	3,835,643.83	528.67
29	--	--	3,903,815	3,903,947.35	552.89	3,917,684	3,917,864.76	543.72
30	--	--	3,991,055	3,991,325.79	574.63	3,999,665	3,999,847.39	567.06
31	--	--	4,078,295	4,078,476.35	603.94	4,097,472	4,097,639.75	593.63
32	--	--	4,165,535	4,165,657.49	624.13	4,174,647	4,174,859.76	610.08
33	--	--	4,252,775	4,252,954.73	633.47	4,263,995	4,264,479.24	619.38
34	--	--	4,296,395	4,296,547.24	656.52	4,307,228	4,307,579.68	641.68
35	--	--	4,340,016	4,342,432.76	671.75	4,361,251	4,371,361.73	642.56

**Table 7:** Objective function values and CPU times of the considered solution approaches.

	ACO			SA		
Example	Objective value	Average value	CPU time (seconds)	Objective value	Average value	CPU time (seconds)
1	271,579	271,599.47	36.38	271,458	272,997.67	36.58
2	352,696	352,796.25	39.17	352,696	352,752.18	38.59
3	451,876	451,953.67	48.39	451,852	451,958.98	47.26
4	505,978	505,9898.69	61.68	505,963	506,656.37	63.48
5	542,468	542,512.58	72.28	542,347	543,546.64	72.49
6	722,784	722,869.47	112.66	722,698	722,883.95	112.68
7	903,769	903,846.91	130.58	903,697	904,681.16	132.23
8	993,638	993,758.36	150.47	993,593	993,759.32	151.15
9	1,094,374	1,094,469.29	159.69	1,094,265	1,107,326.05	156.32
10	1,266,987	1,266,996.47	180.83	1,266,853	1,266,998.34	182.54
11	1,449,997	1,450,038.59	200.47	1,449,995	1,450,162.75	202.95
12	1,637,756	1,637,846.61	218.36	1,637,683	1,637,857.23	218.47
13	1,818,602	1,818,748.97	239.47	1,818,595	1,818,753.65	238.12
14	1,996,768	1,996,884.47	279.04	1,996,663	1,996,866.53	276.56
15	2,098,483	2,098,522.39	297.64	2,098,381	2,098,558.39	298.05
16	2,195,708	2,195,796.34	321.42	2,195,636	2,192,426.82	322.52
17	2,343,693	2,343,743.29	347.58	2,343,589	2,343,695.35	347.53
18	2,512,768	2,512,863.93	367.75	2,512,663	2,512,878.53	367.13
19	2,695,812	2,695,874.47	386.75	2,695,752	2,695,996.32	388.74
20	2,878,986	2,879,148.86	396.69	2,878,956	2,879,153.58	397.58
21	2,999,384	2,999,569.36	409.12	2,999,254	2,999,574.25	402.17
22	3,186,789	3,186,843.97	426.47	3,186,645	3,186,887.57	425.62
23	3,343,318	3,343,476.68	445.12	3,343,186	3,343,357.15	446.81
24	3,499,758	3,499,876.32	459.32	3,499,648	3,499,879.68	457.73
25	3,565,689	3,565,743.68	474.08	3,565,551	3,565,775.27	476.65
26	3,671,247	3,671,358.03	481.85	3,671,142	3,672,291.26	482.42
27	3,736,602	3,736,856.56	508.46	3,736,686	3,736,883.62	509.78
28	3,835,496	3,835,569.39	528.18	3,835,483	3,835,656.48	527.54
29	3,917,697	3,917,805.68	543.42	3,917,695	3,917,876.05	544.15
30	3,999,686	3,999,849.49	566.26	3,999,673	3,999,869.74	568.39
31	4,097,489	4,097,638.35	592.75	4,097,489	4,097,646.38	596.25
32	4,174,663	4,174,869.49	609.75	4,174,651	4,174,868.59	612.35
33	4,264,039	4,264,146.26	620.08	4,263,998	4,264,486.83	618.49
34	4,307,357	4,307,593.38	639.71	4,307,236	4,307,569.63	642.59
35	4,361,369	4,361,409.21	641.86	4,361,268	4,371,375.27	643.78

As for the GA, ACO and SA computational performance, in examples from 1 to 4, the objective function value obtained by PSMBO was smaller than that obtained by GA, ACO and SA (see **Tables 6** and **7**). This is because there is a transportation matrix in the optimal solution of small-scale example 1 as shown in **Table 8**. The priority-based encoding method applied by GA, ACO and SA was not able to obtain such a solution. This underlines a defect of the priority-based encoding method itself. Therefore, for small-scale examples (examples from 1 to 8), the objective function values obtained by PSMBO were superior to the ones obtained by GA. For examples from 9 to 35, the objective values obtained by PSMBO were still smaller than those of GA, ACO and SA, but for all the examples, the running time was longer than that of GA, ACO and SA. With the increase of the problem size, the increase of the time was greater, which is caused by the more time spent for the initial population generation. However, this process, as the core part of the algorithm design, can improve the accuracy of the algorithm search, and is the key to the algorithm’s ability to search for the good-quality solutions. Therefore, although the PSMBO algorithm runs longer than the GA, ACO and SA algorithm, the quality of the PSMBO solutions is higher. The computational time of PSMBO still can be viewed as acceptable from a practical point of view even for large-scale problem examples. Thus, the numerical experiments confirm that PSMBO consistently provides higher quality solutions than those of GA, ACO and SA, which is a common metaheuristic algorithm that has been applied for a large variety of optimization problems in the state-of-the-art.

**Table 8:** Optimal transportation volumes from factories to distribution centers in example 1.

Quantity to be transported	12	5	3	9	6	8
	Distribution center (capacity)					
Factory (capacity)	1(15)	2(10)	3(10)	4(10)	5(10)	6(10)
1(15)	0	0	0	7	0	0
2(20)	0	0	0	2	6	0
3(15)	12	0	3	0	0	0
4(15)	0	5	0	0	0	8

**Fig 3:**
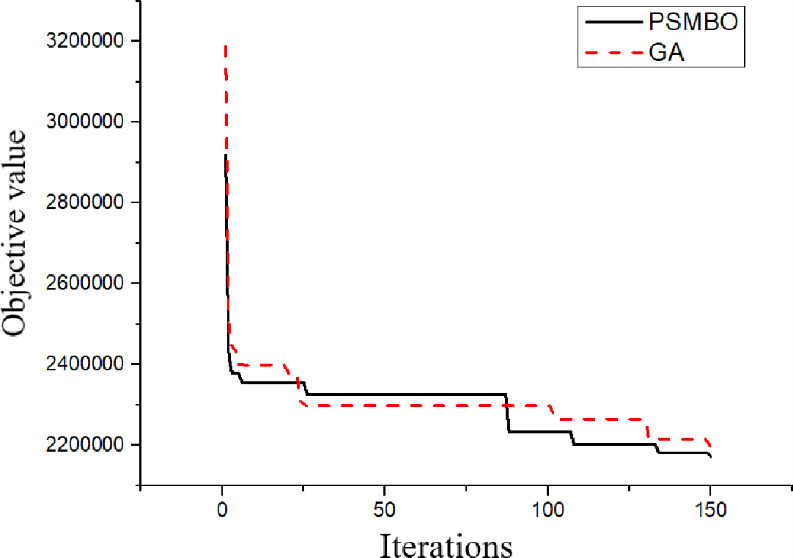
Convergence patterns of PSMBO and GA for example 4.

To better investigate search capabilities of the PSMBO algorithm, PSMBO and GA were used to solve example 16, and the convergence patterns of PSMBO and GA are presented in Fig 3. Based on the results from conducted experiments, it can be seen that under the same conditions and presence of global search and local search operators, the PSMBO algorithm with the product source encoding has obtained better results than the GA algorithm with the priority-based encoding. The proposed product source encoding scheme allowed PSMBO effectively avoiding local optima, especially at early iterations of the algorithm. Furthermore, the product source encoding scheme adopted within the PSMBO algorithm generated superior solutions at the population initialization stage when comparing to the priority-based encoding scheme adopted within the GA algorithm. Therefore, the proposed PSMBO algorithm has clear advantages in terms of its explorative and exploitative capabilities throughout the search process.

### 5.3. Sensitivity analysis and managerial insights

To analyze the impact of fuzzy parameters on the considered closed-loop supply chain network, example 1 was investigated in detail as a part of the computational experiments. The median value of the fuzzy remanufacturing rate was set to 0.9, and the weight of non-remanufactured products per unit was set to 20. The remaining values of required parameters are presented in **Tables 9** and **10**. A total of 11 scenarios were developed by changing the confidence level between 50% and 100% with an increment of 5%, and the PSMBO algorithm was used as a solution approach due to its competitive performance. The analysis results of the closed-loop supply chain network model for each confidence level scenario are shown in **Table 11**.

**Table 9:** Related data for the considered facilities in example 1.

Node	Node number	Midpoint value of the fuzzy capacity	Opening cost (10000)	
				Median value of unit fuzzy purchase cost
	*I*1	200		58
Supplier (*I*)	*I*2	210		60
	*I*3	180		55
	*I*4	240		56
	*I*5	250		57
				Intermediate value of unit fuzzy manufacturing cost
	*J*1	250	23500	480
Factory (*J*)	*J*2	280	23000	460
	*J*3	200	24000	500
	*J*4	320	22500	450
Distribution Center (*K*)	*K*1	270	12000	
	*K*2	250	12500	
	*K*3	210	13000	
	*K*4	220	12500	
	*K*5	300	12000	
	*K*6	260	13000	
	*H*1	287	10000	
Recycling Center (*H*)	*H*2	277	11000	
	*H*3	225	12000	
	*H*4	265	11000	
				Intermediate value of unit fuzzy operating cost
	*R*1	*E*1:260, *E*2:320, *E*3:270, *E*4:250	*E*1:11000, *E*2:10000, *E*3:11000, *E*4:10000	*E*1:30, *E*2:33, *E*3:32, *E*4:31
Disassembly center (*R*) (*E*: Production process)	*R*2	*E*1:200, *E*2:220, *E*3:260, *E*4:320	*E*1:10000, *E*2:11000, *E*3:12000, *E*4:12000	*E*1:33, *E*2:30, *E*3:35, *E*4:35
	*R*3	*E*1:320, *E*2:260, *E*3:320, *E*4:260	*E*1:12000, *E*2:12000, *E*3:10000, *E*4:11000	*E*1:35, *E*2:35, *E*3:30, *E*4:30
	*M*1	150	10000	
	*M*2	250	11000	
Processing center (*M*)	*M*3	280	12000	
	*M*4	170	13000	
	*M*5	200	12000	

**Table 10:** Relevant data for the considered consumer areas.

Consumption area	1	2	3	4	5	6	7	8	9	10
Volume of demand	10	20	10	20	10	20	10	20	10	10
The median value of unit fuzzy purchase cost	230	240	220	230	240	220	200	210	200	210
The median value of unit fuzzy recovery rate	0.58	0.67	0.72	0.83	0.65	0.78	0.82	0.79	0.68	0.81

**Table 11:** Location schemes and objective function values for different values of the confidence level.

Confidence level	$B_{4}^{1}$	$B_{1}^{2}$	$B_{1}^{3}$	$B_{2}^{3}$	$B_{4}^{3}$	$B_{12}^{4}$	$B_{1}^{5}$	$B_{2}^{5}$	$B_{3}^{5}$	$B_{5}^{5}$	Objective value
100%	1	1	0	0	1	1	0	0	1	0	271386
95%	1	1	1	0	0	1	0	1	0	0	265364
90%	1	1	1	0	0	1	0	1	0	0	259243
85%	1	1	1	0	0	1	0	0	0	1	248322
80%	1	1	1	0	0	1	0	0	0	1	243010
75%	1	1	1	0	0	1	1	0	0	0	225388
70%	1	1	1	0	0	1	1	0	0	0	219398
65%	1	1	1	0	0	1	1	0	0	0	213157
60%	1	1	1	0	0	1	1	0	0	0	204762
55%	1	1	1	1	0	1	0	0	0	0	198912
50%	1	1	1	0	0	1	0	0	0	0	175725

In **Table 11**, as a whole, with the increase of confidence level, the location of facilities in the closed-loop supply chain network changes, the total number of facilities generally increases, and the target objective function value increases. Such patterns can be justified by the fact that the increase in confidence level leads to an increase of credible demand, which increases the amount of products transported along the network. To maintain the feasibility of the network, the number of facilities to be opened or the facilities with larger capacity but higher opening costs increases, which increases the fixed opening cost of facilities. At the same time, the transportation cost also increases with the increase in logistics volumes, which ultimately increases the target objective function value.

After a careful analysis of **Table 11**, when the confidence level is 50%~100%, the best solution is to select the candidate disassembly center “2”, factory “4”, distribution center “1”, and operation mode 1. When the confidence level is 100%, the difference between the obtained solution and the solutions with a confidence level of 50%~95% is that the former chooses recycling center “4”, the latter chooses recycling center “1”, and the solution with a confidence level of 55% also chooses recycling center “2”. As for the site selection of the processing center, the processing centers are not selected for the solutions with a confidence level of 50%~55% (i.e. all used products were subject to recycling and then remanufacturing, so there was no need to have a processing center for the used products that cannot be subject to remanufacturing). Processing center “1” was selected for the solutions with a confidence level of 60%~75%, whereas processing center “5” was selected for the solutions with a confidence level of 80%~85%. Moreover, processing center “2” was selected for the solutions with a confidence level of 90%~95%, and processing center “3” was selected for the solution with a confidence level of 100%. The conducted analysis shows that the number of facilities for the solutions with the 50%~100% confidence level remains almost the same, but the locations of facilities changes, that is, the change in the confidence level directly affects the structure of the supply chain network.

**Fig 4:**
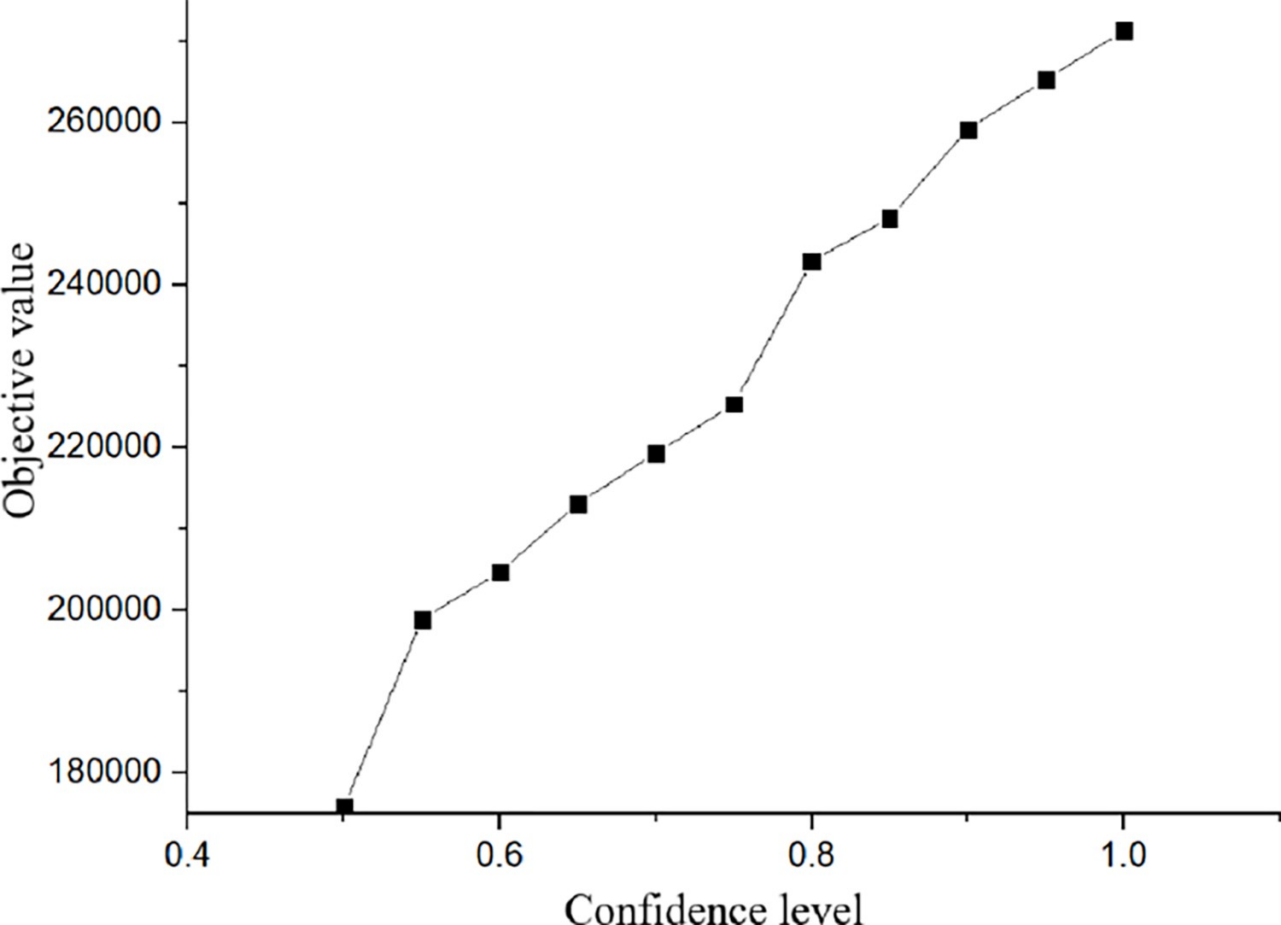
Changes in the target objective function for different confidence levels.

As shown in Fig 4, when the confidence level increases in the range of 90%~100% and 55%~75%, the relationship between the confidence level and the target objective function (cost) value is approximately linearly proportional (that is, the rates of increasing target value are similar for these confidence level scenarios). When the confidence level is between 75%~90% and between 50%~60%, the increase in the confidence level is not proportional to the increase in the cost, and the target value rapidly increases with the confidence level for the scenarios with the confidence levels of 50%~55% and 75%~80%. On the contrary, the increase in the target value for the confidence level range of 80%~85% is not substantial. Therefore, based on the results from the conducted analysis, it can be concluded that the change in the confidence level may drastically change of target cost value, which can be risky for the closed-loop supply chain players. To sum up, the confidence level may substantially affect the structure of the supply chain network. Hence, enterprise managers, in the process of grasping the changes in the current economic situation, must reasonably estimate customer demand and select the appropriate confidence level to bear the adequate level of corresponding risks, and design an efficient closed-loop supply chain network at the strategic level.

Based on the above analysis, the following management insights can be drawn:

- The above analysis suggests that the closed-loop supply chain network optimization model exhibits a high sensitivity to confidence levels. When selecting network facilities, it is necessary to accurately estimate the uncertain parameters within the network. The analysis of example results reveals that as the confidence level increases, the cost budget also increases. Beyond a certain level, the cost budget rapidly increases. Different confidence levels may lead to different optimization designs for closed-loop supply chain networks. Decision makers often need to provide reasonable confidence levels through investigation and research to ultimately determine the optimal design of logistics networks.
- The utilization of fuzzy programming in the logistics recycling network of a closed-loop supply chain can enhance operational efficiency, facilitate rational resource allocation, and offer data-driven support for planning. This approach can enhance the precision and responsiveness of the network, ensuring its stable and reliable operation.
- When the confidence level changes, the feasible region of the optimization model gradually decreases as the confidence level increases, and the optimal value of the model increases as the confidence level increases. Therefore, the optimal value range for the closed-loop supply chain network is between 175,725 and 271,386.
- After decision-makers conduct research to determine a reasonable level of confidence, they can establish the closed-loop supply chain network, achieving the minimum total cost of the closed-loop supply chain logistics system, the number, location, and type of logistics facilities, as well as the logistics volume between facilities and consumption areas.

## Section 6: Conclusion

In recent years, driven by environmental concerns, manufacturing companies have been attempting to reuse and remanufacture their products to reduce pollution caused by product scrapping. Recognizing the substantial economic and environmental benefits that can be achieved from the recycling of used products, the current research presents a facility location optimization problem for closed-loop supply chains. The proposed closed-loop supply chain network consists of suppliers, factories, distribution centers, consumption areas, recycling centers, dismantling centers, and processing centers. In current research, different customers’ demands for products, recovery rates, remanufacturing rates, fixed open costs, variable costs, and transportation costs are assumed to be randomly variable. A Mixed Integer Programming model is proposed to minimize the total cost of the network, including fixed opening cost, operating cost in facilities, procurement cost, collection cost, and transportation cost.

For the current problem is introduced, this study proposed a novel fuzzy closed-loop supply chain network programming model, which relied on the fuzzy ranking method based on a credibility measure. The model took into account the uncertainty of critical supply chain network parameters, including demand, opening cost of facilities, capacity of opened facilities, transportation cost, and procurement cost. Then, according to the characteristics of this model, a new solution encoding method based on the product source encoding was proposed and used in the framework of the Migratory Birds Optimization Algorithm. Finally, several computational examples were used to prove the effectiveness of the algorithm based on a comparative analysis against the exact optimization method (LINGO) and Genetic Algorithm, Ant Colony Optimization, Simulated Annealing, which are recognized as a well-known metaheuristic algorithm. The results from extensive experiments showed that the proposed algorithm was able to provide optimal and good-quality solutions within acceptable computational time even for large-scale numerical examples. The applicability of the model was demonstrated via a sensitivity analysis, which was conducted by changing the confidence levels for the uncertain parameters of the model. The outcomes of this research are expected to provide scientific support for related supply chain enterprises and stakeholders.

The results show that the four traditional encoding methods, including product source encoding and Prüfer number encoding, spanning tree encoding, forest data structure encoding, and priority encoding, are more conducive to finding the optimal solution. Furthermore, when the four traditional encoding methods fail to find the optimal solution, the product source encoding method can all search for the optimal solution. Additionally, based on the product source encoding method, it is easier to escape local solutions. Moreover, the difference in CPU time spent by PSMBO is not much higher than that spent by GA, ACO, and SA. Furthermore, compared to GA, ACO, and SA, the PSMBO algorithm achieves a smaller target value, i.e. the total cost, in different cases. Compared to the changes in total cost observed from the network using GA, ACO, and SA, the total network cost obtained from PSMBO provides a smaller value. This indicates that the PSMBO algorithm based on changes in the total cost of the network achieves better robustness. The current research is of great significance for optimizing the closed-loop supply chain network of certain products with certain demand value and within a certain range of changes. The proposed PSMBO can provide robust results for the optimized network, which can be achieved under different parameter ranges. Furthermore, the confidence level is determined by enterprise managers based on the actual situation, so this study reflects a combination of qualitative and quantitative research methods.

The present research can be expanded further in several dimensions. In the future, we can fully consider the coordinated development of the capacity of the critical nodes (referring to each facility) and edges (referring to the transportation routes) of the supply chain network to fully capture the overall transportation function and improve the service level. In addition, the encoding method proposed in this study can be viewed as general and needs to further deepen its network structure for different scenarios. Furthermore, the proposed product source encoding method can be applied and investigated for other metaheuristic algorithms widely used in the state-of-the-art to expand its application scope.

Research to date has encompassed economic impact considerations in site selection, transportation, and business operations within supply chain networks. However, this body of work has given comparatively less attention to environmental and social decision variables, despite their substantial and distinctive importance, calling for broader investigation. Moreover, while current research encompasses decision variables at strategic, tactical, and operational levels, their integration remains inadequate, particularly with respect to aligning facility location selection, vehicle routing, and inventory control. These three decisions are pivotal in reverse logistics system planning, being interconnected and mutually reinforcing in their impact on the logistics system’s operational effectiveness. Consequently, optimizing the integration of reverse logistics system location, path, and inventory under sustainable objectives represents a significant area for further scholarly exploration. Furthermore, integrating scheduling decisions, pricing strategies, and other critical aspects of reverse logistics into the design of sustainable reverse logistics networks can foster coherence across decision-making tiers and extend the boundaries of existing research.
