# Expression of Concern: An Improved Migratory Birds Optimization Algorithm for Closed- Loop Supply Chain Network Planning in a Fuzzy Environment

**DOI:** 10.1371/journal.pone.0329803

**Published:** 2025-08-06

**Authors:** The PLOS One Editors

After this article [1] was published, concerns were identified by the *PLOS One* Editors regarding the article’s peer review and compliance with the PLOS Authorship policy. We regret that the issues were not addressed prior to the article’s publication.

In light of the cumulative issues, the *PLOS One* Editors issue this Expression of Concern. Readers are advised to interpret the article [1] with caution.
